# CSF1R inhibition depletes brain macrophages and reduces brain virus burden in SIV-infected macaques

**DOI:** 10.1093/brain/awae153

**Published:** 2024-07-25

**Authors:** Diana G Bohannon, Laurent D Zablocki-Thomas, Evan S Leung, Jinbum K Dupont, Julian B Hattler, Jolanta Kowalewska, Miaoyun Zhao, Jiangtao Luo, Marco Salemi, Angela M Amedee, Qingsheng Li, Marcelo J Kuroda, Woong-Ki Kim

**Affiliations:** Department of Microbiology and Molecular Cell Biology, Eastern Virginia Medical School, Norfolk, VA 23507, USA; Department of Anatomy, Physiology & Cell Biology, University California, Davis School of Veterinary Medicine, Davis, CA 95616, USA; Department of Microbiology and Molecular Cell Biology, Eastern Virginia Medical School, Norfolk, VA 23507, USA; Department of Microbiology and Molecular Cell Biology, Eastern Virginia Medical School, Norfolk, VA 23507, USA; Department of Microbiology and Molecular Cell Biology, Eastern Virginia Medical School, Norfolk, VA 23507, USA; Department of Pathology and Anatomy, Eastern Virginia Medical School, Norfolk, VA 23507, USA; Nebraska Center for Virology, School of Biological Sciences, University of Nebraska-Lincoln, Lincoln, NE 68583, USA; Department of Health Systems and Population Health Sciences, the Tilman J. Fertitta Family College of Medicine, University of Houston, Houston, TX 77204, USA; Department of Epidemiology, University of Florida College of Medicine, Gainesville, FL 32610, USA; Department of Microbiology, Immunology & Parasitology, Louisiana State University Health Sciences Center, New Orleans, LA 70112, USA; Nebraska Center for Virology, School of Biological Sciences, University of Nebraska-Lincoln, Lincoln, NE 68583, USA; Department of Anatomy, Physiology & Cell Biology, University California, Davis School of Veterinary Medicine, Davis, CA 95616, USA; Department of Microbiology and Molecular Cell Biology, Eastern Virginia Medical School, Norfolk, VA 23507, USA; Division of Microbiology, Tulane National Primate Research Center, Covington, LA, 70433, USA; Department of Microbiology & Immunology, Tulane University School of Medicine, New Orleans, LA 70112, USA

**Keywords:** BLZ945, CSF1R, HIV, perivascular macrophage, SIV

## Abstract

Perivascular macrophages (PVMs) and, to a lesser degree, microglia are targets and reservoirs of HIV and simian immunodeficiency virus (SIV) in the brain. Previously, we demonstrated that colony-stimulating factor 1 receptor (CSF1R) in PVMs was upregulated and activated in chronically SIV-infected rhesus macaques with encephalitis, correlating with SIV infection of PVMs. Herein, we investigated the role of CSF1R in the brain during acute SIV infection using BLZ945, a brain-penetrant CSF1R kinase inhibitor.

Apart from three uninfected historic controls, nine Indian rhesus macaques were infected acutely with SIVmac251 and divided into three groups (*n* = 3 each): an untreated control and two groups treated for 20–30 days with low- (10 mg/kg/day) or high- (30 mg/kg/day) dose BLZ945. With the high-dose BLZ945 treatment, there was a significant reduction in cells expressing CD163 and CD206 across all four brain areas examined, compared with the low-dose treatment and control groups.

In 9 of 11 tested regions, tissue viral DNA (vDNA) loads were reduced by 95%–99% following at least one of the two doses, and even to undetectable levels in some instances. Decreased numbers of CD163+ and CD206+ cells correlated significantly with lower levels of vDNA in all four corresponding brain areas. In contrast, BLZ945 treatment did not significantly affect the number of microglia. Our results indicate that doses as low as 10 mg/kg/day of BLZ945 are sufficient to reduce the tissue vDNA loads in the brain with no apparent adverse effect. This study provides evidence that infected PVMs are highly sensitive to CSF1R inhibition, opening new possibilities to achieve viral clearance.

## Teaser

Perivascular macrophages are targets and reservoirs of HIV and simian immunodeficiency virus (SIV) in the brain. Bohannon *et al.* show that a brain-penetrant colony-stimulating factor 1 receptor kinase inhibitor depletes brain perivascular macrophages but not microglia and reduces brain viral load in SIV-infected rhesus macaques.

## Introduction

Despite widespread use of suppressive combination antiretroviral therapy (ART), HIV-associated neurocognitive disorder (HAND) remains surprisingly common in people living with HIV.^1-3^ Even in individuals on successful ART with no evidence of plasma virus replication, HAND is still observed.^4-7^ HIV and the closely-related simian immunodeficiency virus (SIV) may persist in the brain ‘sanctuary’, where access of otherwise potent antiretrovirals is limited by the blood–brain barrier (BBB). Proviral DNA remains detectable in the brain, although highly-active ART (HAART) is largely effective at suppressing virus replication in the blood and brain.^8,9^ It is increasingly recognized that HIV RNA may be detected in the CSF of ART-treated patients with undetectable plasma viral loads.^7,10-13^ These findings demonstrate an inability of current treatments to eradicate HIV-infected cells and the persistence of HIV in the CNS despite suppressive ART. The principal challenges to eradicating HIV from the brain include (i) identifying and characterizing reservoirs of HIV persisting in the brain during effective ART; and (ii) delivering therapeutics to HIV reservoirs behind the BBB.

Clements and colleagues recently demonstrated, using an SIV/macaque model of ART viral suppression, that SIV-infected brain macrophages persist during ART.^14,15^ In these studies, the latent virus was reactivated from its myeloid source in the CNS during latency reversal in the presence of suppressive ART. These studies indicate that brain macrophages harbour replication-competent SIV, further demonstrating a high unmet need for therapeutic strategies to specifically target the HIV-infected myeloid cells in the brain.

We have turned to therapeutically exploiting, for HIV brain infection, the inhibitory effects of CNS-penetrant colony-stimulating factor 1 receptor (CSF1R) inhibitors on lesion-associated macrophages and microglia overexpressing CSF1R. Promising preclinical proof-of-concept has been established that these orally bioavailable CSF1R inhibitors can be used to target/block the activation and proliferation of brain-resident macrophages and microglia behind the BBB for the treatment of Alzheimer’s disease.^16-18^ Previously, we found increased proliferation of brain macrophages in HIV and SIV encephalitis, in line with upregulation and activation of CSF1R in the brain, correlating with the severity of SIV encephalitis.^19,20^ In this study, we investigated the role of CSF1R-postive myeloid cells as a persistent cellular target in the brain during acute SIV infection using a CNS-penetrant small molecule CSF1R kinase inhibitor, BLZ945. We demonstrated that BLZ945 treatment depleted brain perivascular macrophages (PVMs) expressing high levels of CSF1R in the brains of acutely SIV-infected macaques. We observed a corresponding significant reduction of SIV DNA viral load in the brains. These data highlight the role of PVMs as a target and potential reservoir of HIV/SIV and demonstrate a previously unrecognized therapeutic benefit of CSF1R blockade.

## Materials and methods

### Animal models and study design

A total of 12 male Indian rhesus macaques (*Macaca mulatta*) between 5 and 8 years of age were used for this study (Supplementary Table 1). Animals were specifically pathogen-free for HIV-2, SIV, type-D simian retrovirus and simian T-cell leukemia virus type 1 at the time of assignment. All procedures involved in this study were approved in advance by the Tulane University Institutional Animal Care and Use Committee (IACUC) and the University of California, Davis IACUC and were carried out in accordance with the National Institutes of Health ‘Guide for the Care and Use of Laboratory Animals’, the recommendations of the Weatherall report, ‘The use of non-human primates in research’, and the ARRIVE (Animal Research: Reporting In Vivo Experiments) guidelines. All primates were socially paired and housed either at the Tulane National Primate Research Center (TNPRC; untreated) or the California National Primate Research Center at UC Davis (CNPRC; treated) in accordance with institutional IACUC regulations and policies under the direction and observation of Dr Marcelo Kuroda at both institutes. Both primate centres are fully accredited by the Association for Assessment and Accreditation of Laboratory Animal Care International. All animals had access to environmental enrichment provided daily, including manipulanda. Supplemental food was provided in addition to commercial chow for non-human primates. All possible measures including analgesics were taken to minimize discomfort of the animals. For all routine procedures such as blood collection and physical examination, animals were fully anaesthetized with ketamine-HCl under the direction of a veterinarian. Animals were humanely euthanized by the veterinary staff at the primate centres in accordance with its end point policy. Euthanasia was conducted by anaesthesia with ketamine-HCl (10 mg/kg body weight) followed by an overdose with sodium pentobarbital. This method is consistent with the recommendation of the American Veterinary Medical Association Guidelines on Euthanasia. Additional archival samples of CSF and plasma from age-matched, sex-matched uninfected Indian rhesus macaques were also used to achieve a baseline for Meso Scale Discovery quantification.

SIVmac251 infects CD4 T cells and tissue macrophages.^21^ It is also known that with experimental CD4 depletion prior to infection, SIVmac251 can infect parenchymal microglia.^22^ SIV infection of nine macaques was achieved through intravenous injection of SIVmac251 and subsequent depletion of CD8 cells with anti-CD8 antibody administered on Days 6, 8 and 12 post infection to ensure consistent viral infection and rapid seeding of virus in the brain.^23^ Of the infected animals, six received a daily oral dose of either 10 or 30 mg/kg of BLZ945 (Novartis) (*n* = 3 each), starting on Day 10 post infection, for 20–30 consecutive days until euthanasia. Animals were randomly grouped. Plasma and CSF were collected at distinct time points throughout the study and tissues were collected post-mortem and frozen or formalin fixed and paraffin embedded (FFPE).

### Immunohistochemistry

FFPE tissues from the frontal cortex, basal ganglia, occipital cortex and cerebellum were sectioned at 5 µm and examined using semi-quantitative singe-label immunohistochemistry (IHC). Briefly, sections were incubated at 60°C overnight before undergoing de-paraffinization and rehydration in a series of xylene and ethanol baths. Citrate- or Tris-based antigen unmasking solution (Vector Laboratories) was applied as a pretreatment in the microwave (1000 W) for 20 min before cooling in solution for an additional 20 min. Slides were then washed using a standard IHC wash buffer of Tris-buffered saline (TBS) with 0.05% Tween-20. Peroxidase block was applied for 10 min before slides were washed and blocked with 5% goat or horse serum with 0.05% Tween-20 for 30 min. After serum was removed, the desired primary antibody was directly applied at the dilutions listed in Supplementary Table 2 at room temperature for 1 h. Following another wash, horse anti-mouse or goat anti-rabbit secondary antibodies were applied at a 1:200 dilution for 30 min. After washing, slides were then treated with Avidin-Biotin Complex (ABC) (Vector Laboratories) for 30 min for amplification purposes and then washed. Diaminobenzidine (DAB) (Vector Laboratories) was applied to each slide for 10 min before undergoing Mayer’s haematoxylin counterstaining, dehydration and mounting.

Brain CD163, CD206 and P2RY12 slides were imaged at ×10 magnification with a Nikon Coolscope digital microscope and quantified using manual counting or ImageJ. Brain CSF1 and liver CD163 and CD68 slides were imaged with a Leica Aperio AT2 digital slide scanner and quantified using Leica Aperio Image Scope 64× and Leica Aperio pixel quantification software to determine the number of positive pixels compared with the total number of tissue-associated pixel per slide. Resulting values were exported to Prism for graphing and statistical analysis.

### Immunofluorescence microscopy

FFPE tissues from the frontal cortex of uninfected and untreated animals were sectioned at 5 µm and examined using semi-quantitative triple-label immunofluorescence (IF) microscopy. Briefly, sections were incubated at 60°C overnight before undergoing de-paraffinization and rehydration in a series of xylene and ethanol baths. Tris-based antigen unmasking solution (Vector Laboratories) was applied as a pretreatment in the microwave (1000 W) for 20 min before cooling in solution for an additional 20 min. Slides were then washed using a standard IF wash buffer of PBS with 0.2% fish skin gelatin. 0.1% Triton X-100 in PBS with 0.2% fish skin gelatin was applied for 1 h to achieve permeabilization before slides were washed and blocked with 5% goat serum with 0.05% Tween-20 for 30 min. After serum was removed, the desired primary antibody was directly applied at the doses listed in Supplementary Table 2 at room temperature for 1 h. Following another wash, isotype specific horse anti-mouse Alexa Fluor 488 or 594 secondary antibodies were applied at a 1:500 dilution for 1 h before being rinsed and subsequent antibodies applied similarly. After removal of the final secondary antibody, DAPI nuclear stain was applied for 5 min before washing and autofluorescence quenching with copper sulfate for 45 min. Finally, slides were washed in water baths and mounted using aqueous mounting media.

IF slides were imaged using a Zeiss Axio Observer.Z1 using a 40× air objective and 1 µm *z*-stack sectioning. Image analysis was performed using the Zen Blue Colocalization Analysis software package by assigning restrictive thresholding to both CSF1R and phospho-CSF1R (p-CSF1R) immunoreactivity and recording the mean pixel intensity (MPI) of p-CSF1R, which co-localized to positive thresholder CSF1R staining in each *z*-stack section of each captured frame. p-CSF1R MPI was then averaged across each *z*-stack section of each captured frame and the average MPI of p-CSF1R immunoreactivity that co-localized to CSF1R positive cells was averaged for each animal cross 10 recorded frames. The average MPI for each animal was then exported into Prism for graphing and statistical analysis.

### Multiplex cytokine assay

The Meso Scale Discovery electrochemiluminescence multiplexing platform was used to evaluate changes in CSF1 levels in the serum and CSF of uninfected, untreated, low-dose and high-dose treated macaques. Pre-infection, post-infection pretreatment, 10 days post-treatment and 30 days post-treatment time points were examined where applicable. The resulting normalized cytokine protein counts were exported for graphing and statistical analysis.

### Viral load quantification

Plasma and CSF RNA viral loads were determined by performing quantitative-reverse transcription (qRT) PCR on plasma and CSF samples from rhesus macaques with a limit of detection of 25 copies/ml. Similarly, brain tissue SIV DNA loads were obtained by performing qPCR on fresh-frozen brain samples from SIV-infected animals at a limit of detection of 25 copies per 1 000 000 cells. Tissue SIV DNA (vDNA) loads were then log transformed and exported into Prism for graphing and statistical analysis. Additionally, SIV RNA (vRNA) was investigated by RNAscope *in situ* hybridization. Briefly, vRNA was detected *in situ* using the RNAscope 2.5 HD-RED Assay [Advanced Cell Diagnostics (ACD)] on 6-μm thick FFPE sections of brain tissues as previously described.^24^ Sections were deparaffinized and rehydrated in xylenes and 100% ethanol and were air dried. Antigen retrieval with boiling citrate buffer for 15 min and protease digestion at 40°C for 30 min was performed. SIV RNA-specific anti-sense probes targeting SIVmac239 envelope (ACD), RNAscope positive control Mmu-PPIB (ACD) or RNAscope negative control DapB (ACD) were applied to sections. Stained tissue sections were scanned and digitized with an Aperio CS2 ScanScope. The number of SIV RNA+ cells from three whole tissues sections of each brain region were manually counted and calculated. Three sections from each area were averaged.

### NanoString nCounter mRNA analysis

Cortical 5-µm FFPE sections (four per animal) from uninfected, untreated, low-dose and high-dose animals were digested with a Qiagen FFPE RNeasy Kit using Qiagen Deparaffinization Solution. Samples were then run on a NanoString nCounter Flex with the non-human primate (NHP) Immunology Panel and the normalized data were exported to Microsoft Excel for further analysis. First, the normalized CSF1R and CSF1 mRNA counts for uninfected and untreated animals were exported to Prism for graphing and statistical analysis. Subsequently, low- and high-dose groups were compared with the untreated group to produce a fold-change ratio and *P*-value for all measured analytes. Microsoft Excel was then used to identify and eliminate any analytes in either comparison which did not meet significance cut-off values of ±1.25 fold-change and a *P*-value <0.05. The significantly differentially regulated analytes are listed in Supplementary Table 3. Individual animal values for differentially regulated analytes were then exported to Prism to determine whether they correlated to CSF1 or CSF1R mRNA values. Analytes that met the criteria to be considered significantly differentially regulated and correlated to either CSF1 or CSF1R mRNA values were then exported to Qiagen Ingenuity Pathway Analysis (IPA) software for further characterization.

Thirty analytes identified by the NanoString NHP Immunology panel were moved into IPA. Of those, 14 analytes were identified as belonging to a distinct pathway demonstrating BLZ945 downregulation of CSF1R. Other markers did not show known interactions with other selected analytes according to the IPA pathway connection analysis or were clustered into small, isolated networks which did not associate with the central pathway relating to the downregulation of CSF1R by BLZ945. While it is possible that these analytes may have a connection to the pathway through an intermediate eliminated from consideration either by the scope of the panel or due to an analyte that did not pass our selection protocols, all such analytes were eliminated from the IPA pathway map to conserve the analytes and pathways most differentially regulated under BLZ945 treatment. These eliminated analytes are pending further review upon completion of our upcoming funded chronic BLZ945 treatment study.

### Liver histological grading

A chemistry analyser (Beckman Coulter AU480) was used to measure serum chemistry from all groups for toxicology and safety testing purposes. Liver histological grading was performed by a board-certified pathologist (J.K.) experienced in renal and hepatic toxicology assessments. Non-alcoholic fatty liver disease (NAFLD) and Ishak liver scoring were performed to identify evidence of liver damage according to the methods described by Ishak *et al.*^25^ and Kleiner *et al.*^26^

### Graphing and statistical analysis

Graphing and statistics were performed under the observation of a PhD statistician (J.L.). GraphPad Prism 9 was used to graph and determine the statistical power of the tests performed. NanoString data were additionally analysed with IPA to create pathway maps.

## Results

### Acute SIV infection results in the upregulation of CSF1R

**Figure 1:**
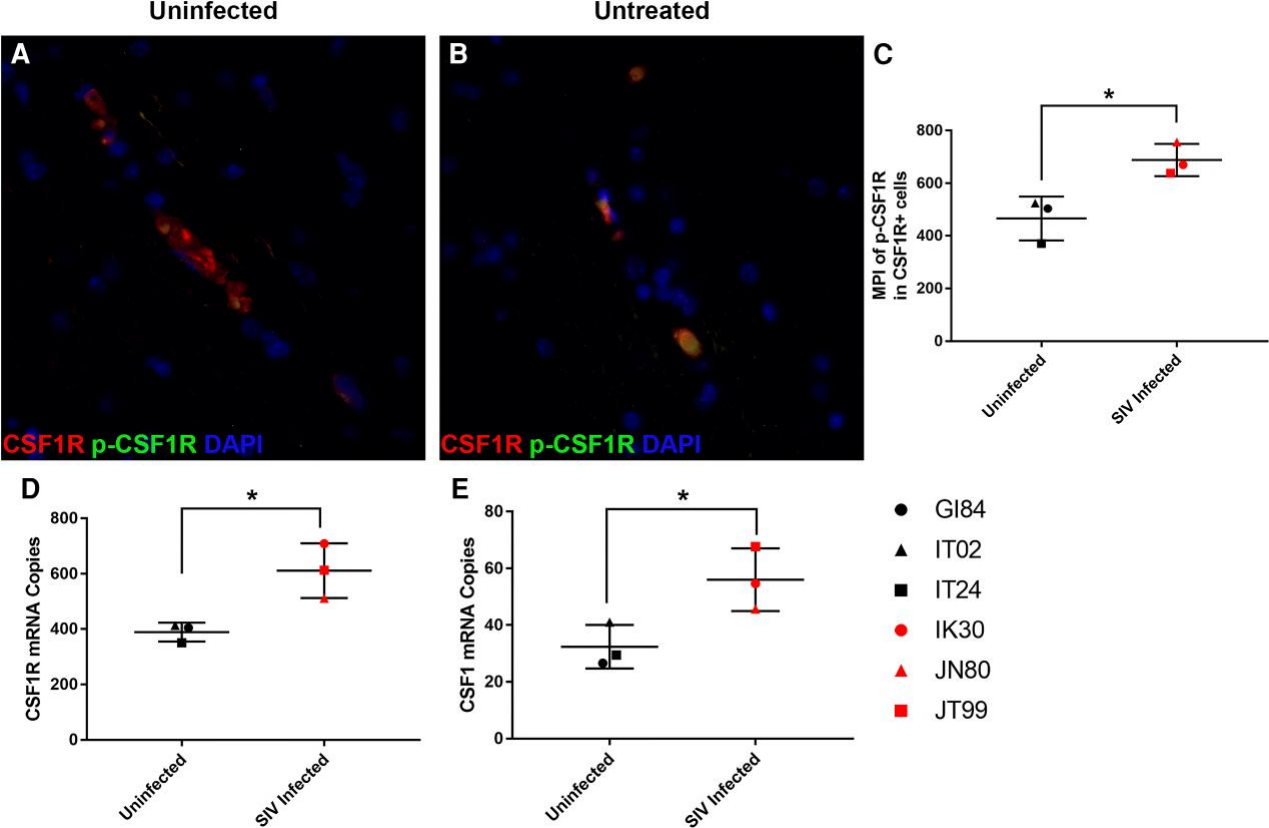
**Phosphorylation activation of CSF1R is increased with simian immunodeficiency virus.** Tri-colour immunofluorescence for CSF1R (red) phosphorylated-CSF1R (p-CSF1R) (green) and DAPI (blue) in the brains of uninfected (**A**) and simian immunodeficiency virus (SIV)-infected animals (**B**) shows an increased mean pixel intensity (MPI) of p-CSF1R in infected animals (red symbols) (**C**). Student’s *t*-test, unpaired, *P < 0.05. Additionally, *CSF1R* (**D**) and *CSF1* (**E**) mRNA were elevated in infected animals compared with uninfected animals (black symbols). Student’s *t*-test, unpaired, *P < 0.05.

To determine if CSF1/CSF1R is upregulated and activated in the brain during acute SIV infection, we examined the expression of CSF1R and its tyrosine phosphorylated activated form (p-CSF1R) in the brain of rhesus macaques infected with SIVmac251 for 22–37 days. We observed increased activation of CSF1R compared with uninfected animals as measured by increased co-localization between CSF1R and p-CSF1R (Fig. 1A–C). In addition, we found that the *CSF1R* mRNA count increased in the cortical tissue of acutely SIV-infected animals compared with uninfected controls (Fig. 1D). Likewise, a corresponding increase in CSF1 protein and mRNA levels was found in comparison with uninfected controls (Fig. 1E). Collectively these results demonstrated that acute SIV infection increases the activation of CSF1R, further demonstrating its potential utility as a therapeutic target.

### CSF1R inhibition decreases macrophage counts throughout the brain

**Figure 2:**
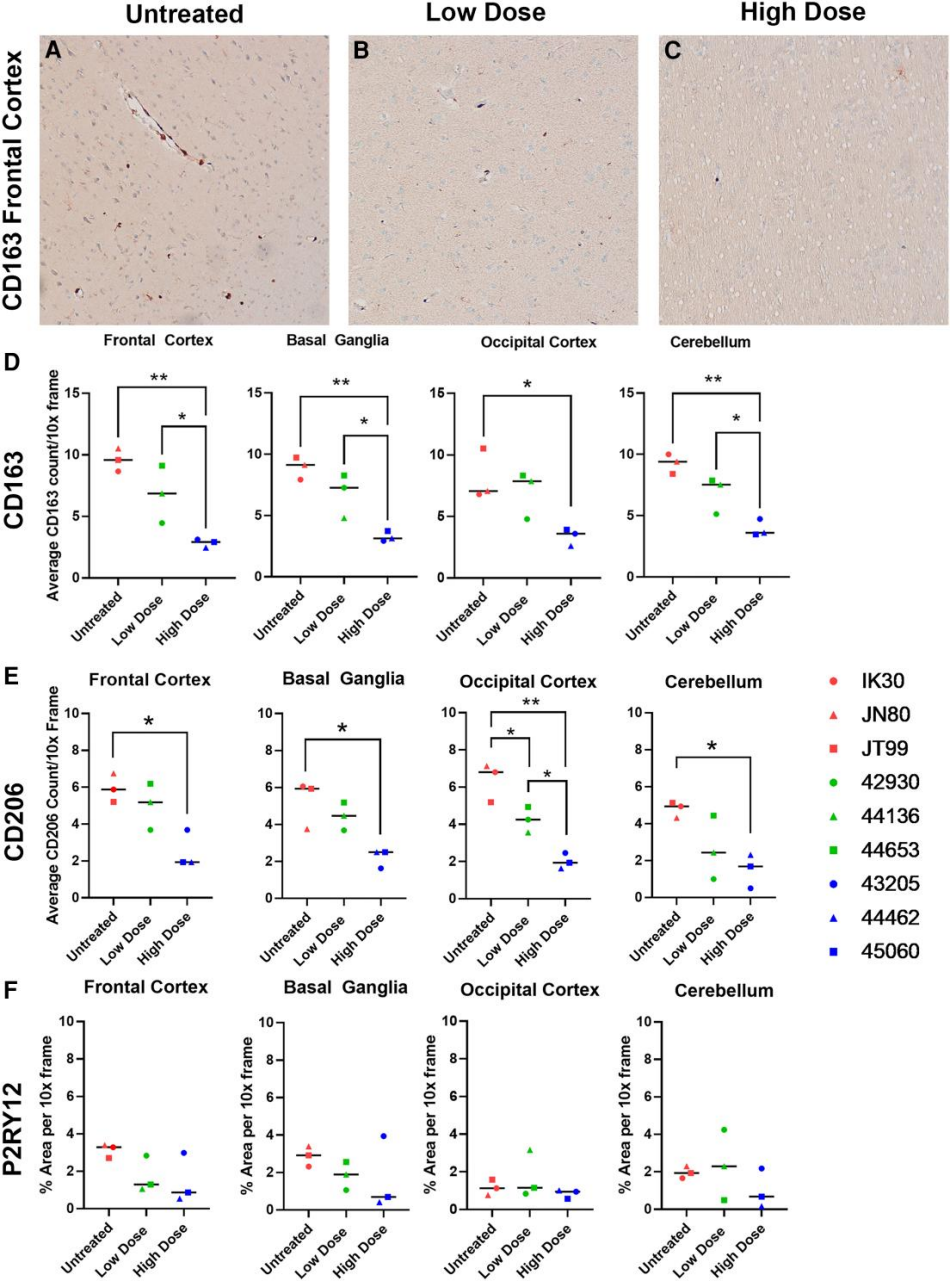
**Macrophages, but not microglia, are reduced after BLZ945 treatment.** Representative images of CD163+ macrophages in the frontal cortex of untreated animals (**A**), and those treated with a low dose (**B**) or high dose (**C**) of BLZ945 show a visual reduction in positive-stained cells corresponding to treatment. Quantification of the average CD206 count per ×10 magnification frame for CD163 cells (**D**), CD206 cells (**E**) and P2RY12 cells (**F**) shows a decrease in CD163+ and CD206+ macrophages, but not P2YR12+ microglia, with treatment. One-way ANOVA with Tukey’s *post hoc* test, *P < 0.05, **P < 0.01. Red symbols = infected and untreated animals; green = infected and low-dose treated animals; blue = infected and high-dose treated animals.

Based on evidence that increased CSF1R activation is important in macrophage survival during SIV/HIV infection, we sought to examine the effects of inhibiting CSF1R during acute SIV infection of rhesus macaques.^27,28^ A highly selective, brain-penetrant CSF1R kinase inhibitor, BLZ945, was orally administered daily starting from 10 days after SIVmac251 infection until termination of the study at 30 to 40 days post-infection. Animals in the low-dose group (*n* = 3) received 10 mg/kg per day, while animals in the high-dose group (*n* = 3) received 30 mg/kg per day. Post-mortem brain tissue was collected and analysed by IHC to evaluate the numbers and phenotypical changes of PVM and microglia populations. The numbers of both CD163+ and CD206+ PVMs were reduced after high-dose BLZ945 treatment in all brain regions examined and reduced in several brain regions of the animals treated with low-dose BLZ945 (Fig. 2A–E and Supplementary Fig. 1A and B). These findings are consistent with a previous *in vitro* study that revealed reduced CD163 expression in tumour-associated macrophages upon treatment with BLZ945.^29^ Notably, in contrast to the results of previous studies,^16,30^ microglia, which express low levels of CSF1R, were not depleted by BLZ945 at the two doses (Fig. 2F and Supplementary Fig. 1C). This discrepancy is likely due to lower doses of BLZ945 being used in this study, but the well-known differences in microglia populations between mice and primates may account for the discrepancy as well.^31,32^ Collectively, these data confirm that BLZ945 can selectively deplete PVMs while not affecting resting microglia, which are essential to maintaining a healthy neuroimmune environment.

### CSF1R inhibition reduces viral load in brain tissue but not plasma or CSF

**Figure 3:**
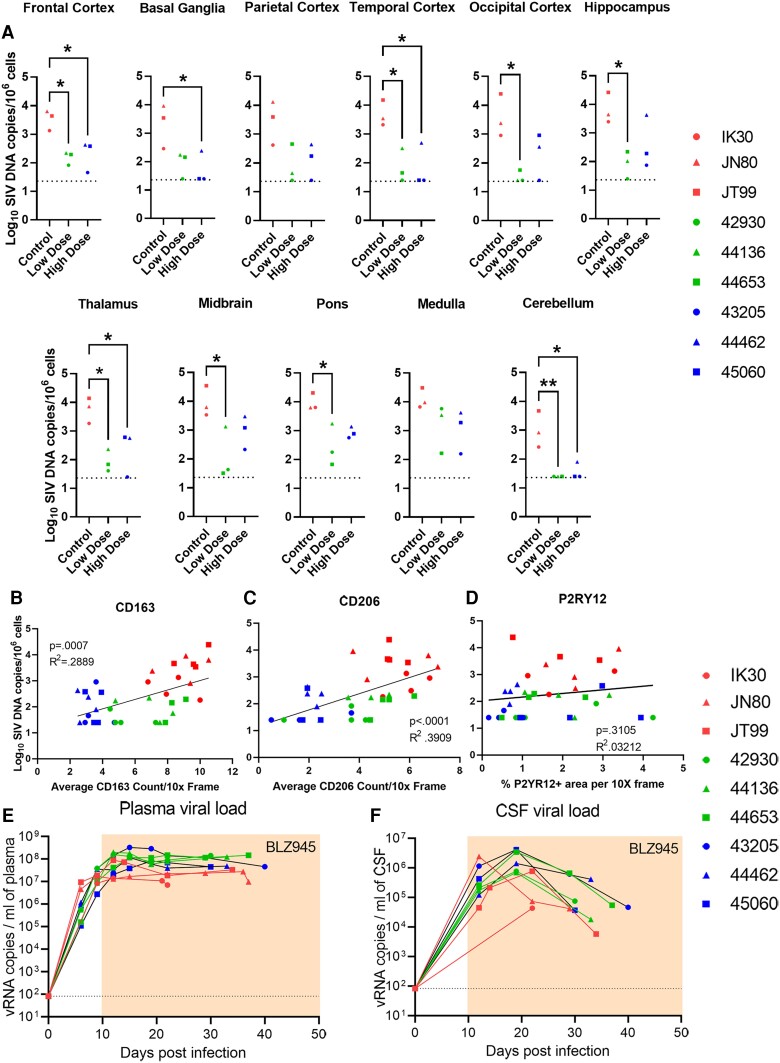
**Brain viral DNA levels decrease in BLZ945-treated animals in correspondence with macrophage loss.** Graphs depicting the simian immunodeficiency virus (SIV) DNA copies per 10^6^ cells as measured by PCR in 11 brain areas depict a decrease in viral load in many brain areas in treated macaques (**A**). One-way ANOVA with Tukey’s *post hoc* test, *P < 0.05, **P < 0.01. The decrease in viral DNA copies is significantly positively correlated with loss of CD163 (**B**) and CD206 (**C**) macrophages but not with P2RY12 (**D**) microglia. Pearson’s correlation. Plasma viral loads (**E**) and CSF viral loads (**F**) showed no measurable effects from treatment. Area under the curve analysis with DeLong’s significance analysis. Red symbols = infected and untreated animals; green = infected and low-dose treated animals; blue = infected and high-dose treated animals.

Having shown that BLZ945 selectively depletes PVMs in the brain, we sought to determine whether depletion of PVMs resulted in a decrease in the associated brain tissue viral loads. We performed qPCR to determine the numbers of vDNA copies in 11 brain regions from untreated animals and animals treated with low- or high-dose BLZ945 (Fig. 3A). In 8 of 11 brain regions of animals treated with a low dose of BLZ945, a significant decrease in vDNA was observed, with levels being undetectable in some regions (Fig. 3A). Animals treated with a high dose of BLZ945 showed a significant decrease in brain tissue vDNA in five regions (Fig. 3A). Additionally, we performed RNAscope to detect vRNA in five brain areas and confirmed that animals treated with a high dose of BLZ945 had virtually no vRNA in all brain regions examined (Supplementary Fig. 2 and Supplementary Tables 4 and 5). Collectively, these results strongly supported a significant reduction in brain viral burden in animals treated with a high dose of BLZ945. Plotting CD163, CD206 or P2RY12 counts to the SIV DNA copy number per million cells for each animal in each brain area showed a significant positive correlation between the brain vDNA levels and PVM numbers but no significant correlation with the number of microglia (Fig. 3B–D and Supplementary Fig. 3A–C). Interestingly, while BLZ945 administration reduced the brain tissue viral burden, all three group of animals showed similar plasma and CSF viral loads, with a peak viraemia around 12 days post-infection for plasma and 20 days post-infection for CSF before stabilizing in the same range of set point values (Fig. 3E and F). Our findings that BLZ945 treatment did not affect plasma or CSF viral loads suggested that during acute infection infected PVMs are not a major source of HIV/SIV RNA in either plasma or CSF.

### BLZ945 is not associated with liver injury in SIV-infected rhesus macaques

**Figure 4:**
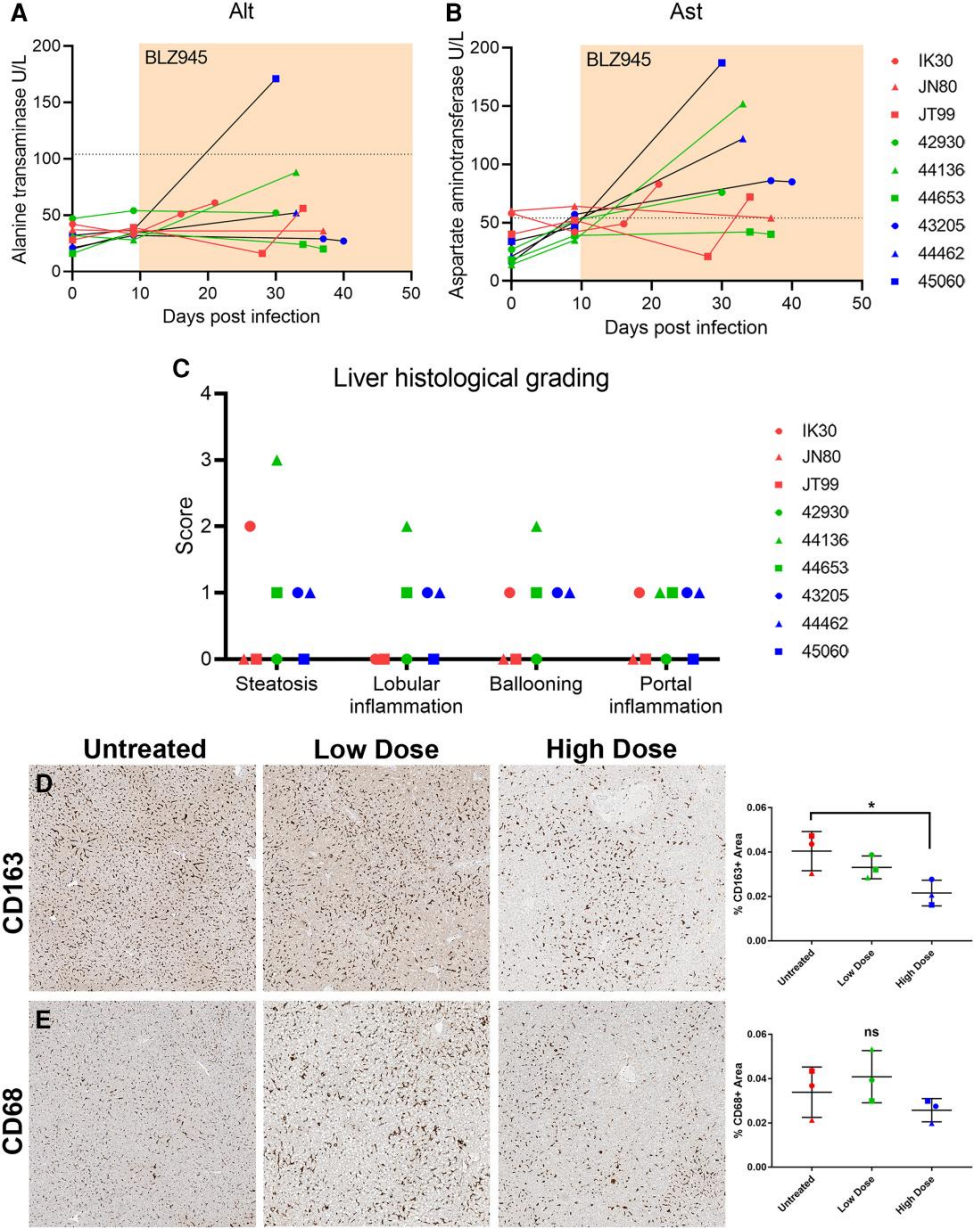
**Liver toxicity was not observed with BLZ945 treatment despite liver macrophage depletion.** Alanine transaminase (ALT) (**A**) and aspartate transaminase (AST) (**B**) levels were measured from pre-infection to necropsy and some treated and untreated animals showed significant elevation in AST above baseline. Area under the curve analysis with DeLong’s significance analysis. Histology scoring however found that there were no significant pathological signs of toxicity in treated animals compared with untreated animals (**C**). Two-way ANOVA with Sidak’s multiple comparison test. Representative images of liver sections stained for CD163 (**D**) or CD68 (**E**) show a significant loss of CD163+ macrophages in high-dose treated animals compared with untreated animals but no significant differences in total CD68+ macrophages. One-way ANOVA with Tukey’s *post hoc* test, *P < 0.05. Red symbols = infected and untreated animals; green = infected and low-dose treated animals; blue = infected and high-dose treated animals.

CSF1R kinase inhibitors and anti-CSF1R antibodies have been associated with potential liver damage, as indicated by elevated blood levels of two liver enzymes: alanine transaminase (ALT) and aspartate transaminase (AST). We investigated whether hepatotoxicity occurred with BLZ945 administration. Moderate increases in serum ALT levels were observed in treated animals but were not significant when compared with untreated controls (Fig. 4A). While all animals treated with a high dose of BLZ945 had higher AST levels, above the healthy range, AST values were elevated after SIV infection regardless of treatment status and the increases seen in treatment groups were not significant when compared with untreated controls (Fig. 4B). To ensure that the observed elevation in AST levels was not an indicator of liver toxicity, NAFLD and Ishak liver scoring were performed to identify evidence of liver damage. Upon histological examination, periportal or periseptal interface hepatitis (piecemeal necrosis), confluent necrosis, focal (spotty) lytic necrosis, apoptosis, focal inflammation, architectural changes, fibrosis and cirrhosis were not found in any animals, and the reported incidences of steatosis, lobular inflammation, ballooning and portal inflammation were non-significant (Fig. 4C). Some reports have suggested that changes in AST and ALT after administration of a macrophage depleting drug may be due to a reduction in chemical recycling efficiency in the liver, which is normally carried out by liver-resident macrophages known as Kupffer cells.^33,34^ We therefore sought to determine whether there was a significant depletion of liver macrophages in BLZ945-treated groups, which may explain the observed elevation of ALT and AST levels with treatment. We examined the number of CD163- and CD68-positive macrophages in the liver and found a significant decrease in the number of CD163+, but not CD68+, cells (Fig. 4D and E). Since CD163 is specific for macrophages in the liver, but CD68 can mark different types of myeloid cells, these findings confirmed that BLZ945 selectively eliminates macrophages in the liver without disrupting other myeloid cell populations. Taken together, these results suggested that, while BLZ945 is effective at targeting CD163+ macrophages in the liver, this activity is not acutely toxic to the liver at the doses tested.

### CSF1R inhibition alters the acute SIV neuroimmune profile

**Figure 5:**
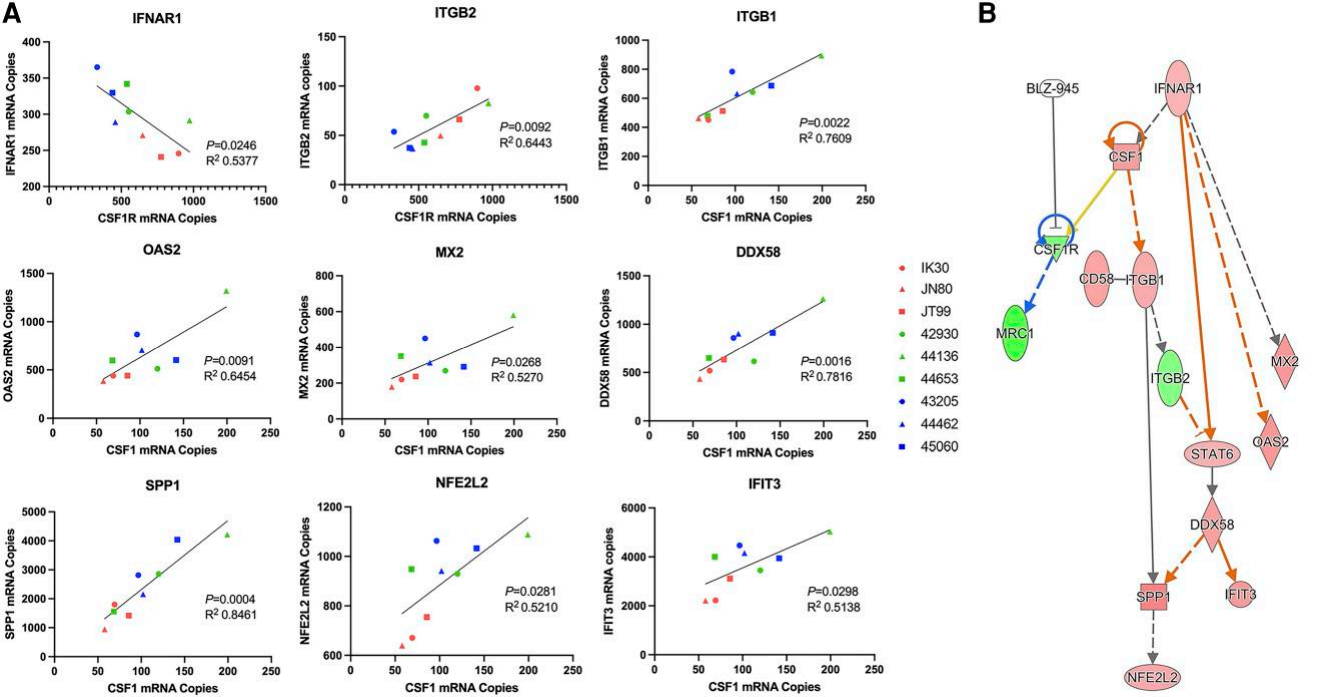
**Treatment with BLZ945 shows reactivation of the interferon viral response pathway.** Selected differentially regulated genes from the NanoString NHP Immunology panel show direct correlations to changes in CSF1 or CSF1R (**A**) and when mapped show that inhibition of CSF1R by BLZ945 may induce upregulation of the IFNAR1 antiviral cascade (**B**). Pearson’s correlation. Red symbols = infected and untreated animals; green = infected and low-dose treated animals; blue = infected and high-dose treated animals.

To investigate the neuroimmune profile, which may be altered in acute SIV infection after oral administration of BLZ945, we measured the mRNA levels of 770 immune response genes, including CSF1 and CSF1R, using the NanoString platform and nCounter NHP Immunology V2 panel. By examining the statistical correlations between changes in CSF1R or CSF1 levels and differentially expressed genes, we found 30 significantly correlated analytes (Fig. 5 and Supplementary Table 3), many of which were antiviral host-immune response factors and found to be increased in BLZ945-treated groups. Of note, IFNAR1, ITGB1, DDX58, IFIT3, NFE2L2, MX2 and OAS2 were significantly upregulated in BLZ945-treated animals compared with untreated controls, suggesting that BLZ945 treatment may reactivate the innate immune system’s ability to target and eliminate SIV in the brain (Fig. 5A). Other significantly correlated analytes showed further evidence of macrophage cell death, reduction of neuroinflammation and a reduction in markers associated with peripheral immune cell infiltrates in BLZ945-treated animals (Supplementary Table 3). Mapping of these differentially expressed and correlated genes by IPA showed that IFNAR1 and its downstream effectors were up-regulated in association with BLZ945-induced inhibition of CSF1R (Fig. 5B). In addition, we observed that a high dose of BLZ945 led to an increase in soluble CSF1 in the CSF, but not in serum, further supporting the role of CSF1 signalling in the brain during viral recovery after treatment with a high dose of BLZ945 (Supplementary Fig. 3D and E). Ultimately, these data suggested that BLZ945 may induce changes in the neuroimmune environment that are advantageous for viral clearance and reduce neuroinflammation often associated with HAND observed in chronically HIV-infected patients on ART.

## Discussion

In the current study, we sought to investigate the role of CSF1R+ brain myeloid cells during acute SIV infection in non-human primates by treating acutely infected rhesus macaques with BLZ945, a small-molecule, brain-penetrant inhibitor of CSF1R. We first established that CSF1R is upregulated and activated in the brain during acute SIV infection, highlighting its value as a potential therapeutic target. Since the activation of CSF1/CSF1R signalling with SIV infection has been suggested as one of the primary mechanisms by which SIV/HIV infected macrophages counteract the host cell’s antiviral apoptotic response to detrimental viral loads, we hypothesized that inhibition of the CSF1/CSF1R axis would lead to increased macrophage death and a subsequent decrease in brain tissue viral loads.^28^ We demonstrated that inhibition of CSF1R kinase with BLZ945 resulted in a depletion of PVMs but not microglia in the brain, which significantly correlated with a decrease in brain tissue viral loads in those regions. While oral administration of BLZ945 at 10 mg/kg or 30 mg/kg for 20–30 days raised liver enzymes, no signs of liver toxicity were observed on post-mortem pathological examination. Finally, our data suggested that administration of BLZ945 reduces the inflammatory profile associated with acute SIV infection and increases factors associated with innate immune driven viral suppression. Collectively, the findings in this study suggested that BLZ945 is safe and effective at lowering the brain tissue viral load during acute SIV infection in rhesus macaques.

Pharmacological targeting of CSF1R either by small molecule inhibitors or neutralizing antibodies has been shown to be beneficial in several animal models of neoplastic and inflammatory diseases. Several of the inhibitors have been tested in clinical trials for rheumatoid arthritis and cancer.^35,36^ The therapeutic effects of CSF1R inhibition are directed at CSF1R-expressing macrophages. Relatively short term (2–4 weeks) treatment with CSF1R inhibitors results in a significant depletion of Ly6C^low^ monocytes (equivalent to human non-classical CD16+ monocytes) and M2-polarized tissue and tumour-associated macrophage subpopulations that express CSF1R at higher levels. It has been shown recently that brain penetrant CSF1R inhibitors have the potential to treat various neurological diseases and brain tumours effectively by directly targeting CNS myeloid cells. Interestingly, while CSF1R inhibitors that target CSF1R and other receptor tyrosine kinases like FLT3 and c-kit are known to deplete even resting ramified microglia rapidly in normal adult brain expressing low amounts of CSF1R possibly due to off-target effects,^16^ treatment with monospecific CSF1R inhibitors, such as GW2580 (PLX6134) and BLZ945, causes selective depletion of activated/proliferating microglia/macrophages that are associated with plaques/lesions and glioma (presumed to be CSF1R^high^).^17,18,37,38^ We have noted that the safety and efficacy of BLZ945, developed by Novartis, has already been tested in cynomolgus macaques,^39^ and it is being tested in humans for the treatment of advanced solid tumours (ClinicalTrials.gov Identifier: NCT02829723). Our previous study in rhesus macaques indicated that PVMs express much higher levels of CSF1R than resting ramified microglia and provided the first *in vivo* evidence that HIV/SIV infection of the CNS induces further upregulation and activation (measured by phosphorylation of CSF1R and STAT5) of CSF1R in PVM and activated microglia but not in resting microglia.^20^ We assumed that pathological activation of CSF1R signalling in infected macrophages would make the cells more vulnerable to apoptosis with CSF1R inhibitor. In this study, we directly demonstrated the contribution of CSF1R^high^ myeloid cells to neuroinvasion of virus during the acute phase (the first month) of infection by eliminating CSF1R^high^ PVMs and activated microglia while sparing CSF1Rlow resting microglia after peak viraemia.

Some findings in this study appear to contradict those from previous rodent studies utilizing BLZ945. One such instance is no apparent loss of microglia in macaques treated with either low- or high-dose BLZ945. In one study, inhibition of CSF1R with 7 and 10 mg/kg doses of BLZ945 using a mouse model of demyelination significantly decreased Iba1 staining in multiple brain regions.^30^ It is, however, important to recognize that Iba1 can stain PVMs as well as microglia in mice and, to a lesser degree, primates, making it an ineffective marker to distinguish microglia from PVMs and assess their relative depletion.^40^ We therefore opted to use P2RY12 as a specific marker for microglia, as we have previously shown that P2RY12 is equally as effective as Iba1 at identifying microglia in macaque brains but does not mark Iba1+ macrophages.^41^ In another study, co-staining of Iba1 with macrophage-specific markers to differentiate between microglia and macrophages showed microglial depletion in mice with 200 mg/kg of BLZ945 administered at postnatal Days 2, 4, 6 and 7.^42^ While microglia progenitors reach seeding saturation in the brain as early as embryonic Day 9, microglia in the mouse brain do not achieve a transcriptional profile similar to that of mature adult microglia until postnatal Day 30 with significant differences between 7 and 30 days. Taken together, this suggested that the microglia being targeted in this study are immature and more attuned to axon guidance (‘activated’) than immune surveillance (‘resting’).^43^ Alternatively, due to CSF1R being expressed at low levels on ‘resting’ adult microglia, higher doses such 200 mg/kg may have been required to induce microglia ablation in adult macaques as seen in mouse studies.

There have been some concerns regarding the potential hepatotoxicity of CSF1R inhibitors based on observed elevation of the liver enzymes ALT and AST. In the current study, we did not observe any evidence of liver toxicity in BLZ945-treated macaques in comparison to untreated animals. While AST levels were elevated in all animals, the increase in enzyme levels observed was most likely due to the depletion of Kupffer cells and the corresponding reduction in the clearance capacity of the liver as suggested by others.^33,34^ In fact, studies have shown that the correlation between ALT and AST levels and liver toxicity is not reliable in multiple cases where the therapeutic being tested targets macrophages or monocytes.^39^ Ultimately, while it will be important to continue to monitor liver health and other signs of toxicity in clinical trials, our results did not indicate any reason why efforts using BLZ945 for the treatment of HIV CNS infection should be discontinued solely based upon increased liver enzymes.

The purpose of this study was to determine the safety and efficacy of BLZ945 as a potential therapeutic for targeting viral reservoirs in the brain and to determine the ideal dose for continued trials. For this purpose, only a small number of animals were treated over a short time span. Despite the limited scope of this study, we succeeded in demonstrating the therapeutic potential of BLZ945 in SIV infection and established that no evidence of unacceptable toxicity was observed. Ultimately, however, a chronic study on a larger cohort will be necessary to demonstrate clinical value. Future studies will focus on the efficacy of BLZ945 to reduce the brain viral burden in chronically SIV-infected animals on ART. Future studies will also examine the ability of BLZ945 to eliminate brain viral reservoirs associated with viral rebound after ART cessation and determine whether treatment can control brain viral burden sufficiently to eliminate the virus at the end of the treatment period. Successful elimination of macrophage viral reservoirs in chronic ART studies in macaques would be a strong indicator to test the efficacy of BLZ945 in combination with ART for the clinical treatment of HIV in the brain.

## Supplementary Material

awae153_Supplementary_Data

## Data Availability

The data presented in this study are available from the corresponding author upon reasonable request.
