# Supplementary material for: CSF1R inhibition depletes brain macrophages and reduces brain virus burden in SIV-infected macaques
Source: Brain. 2024 Jul 25;147(9):3059–69. doi: 10.1093/brain/awae153 (PMC11370798; doi:10.1093/brain/awae153)
Supplement: awae153_Supplementary_Data [file awae153_supplementary_data.zip › brain-2023-01175-File007.pdf]

Supplementary material

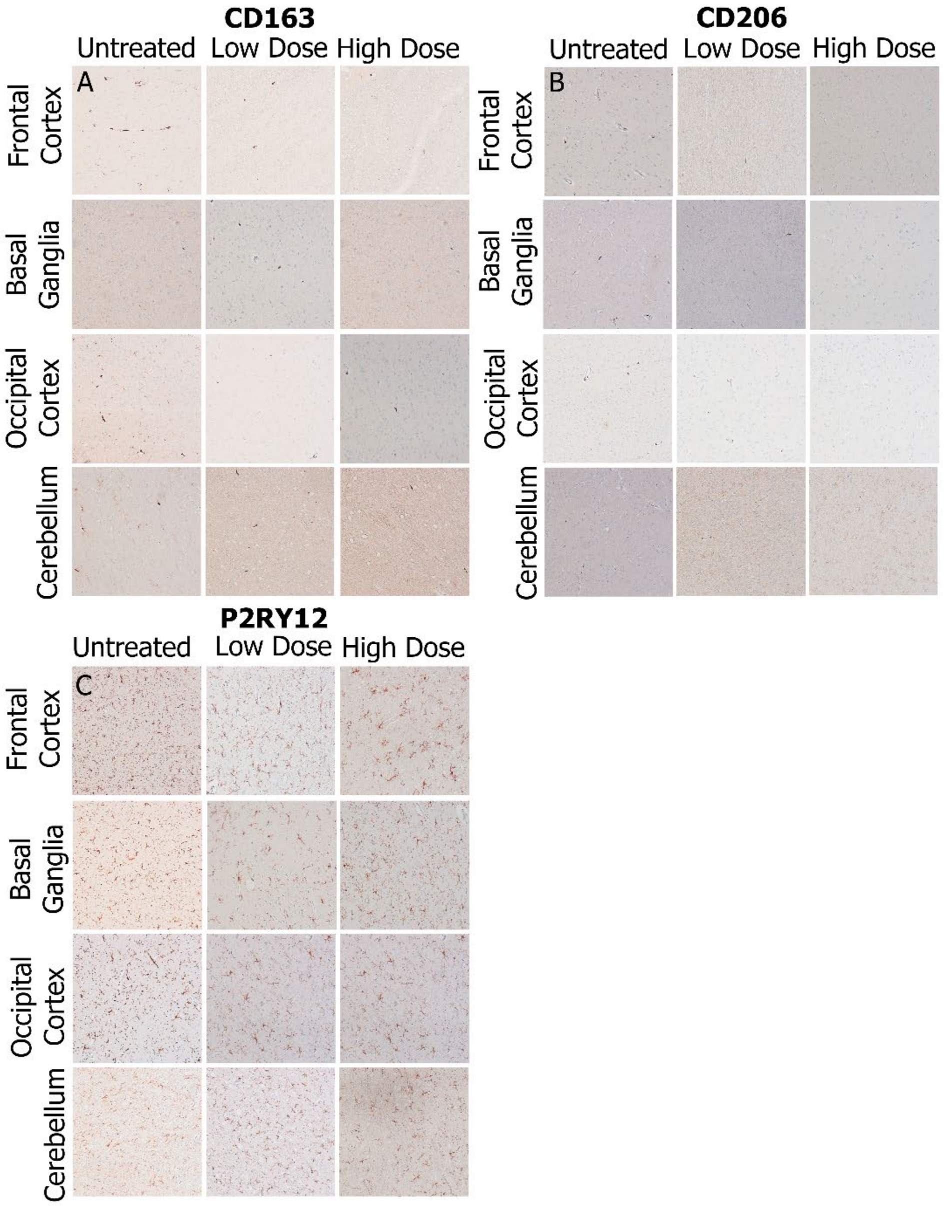

Supplementary Figure 1. Histological staining of brain tissue.
Representative images of CD163 (A), CD26 (B), and P2RY12 (C) from each animal in each brain area.

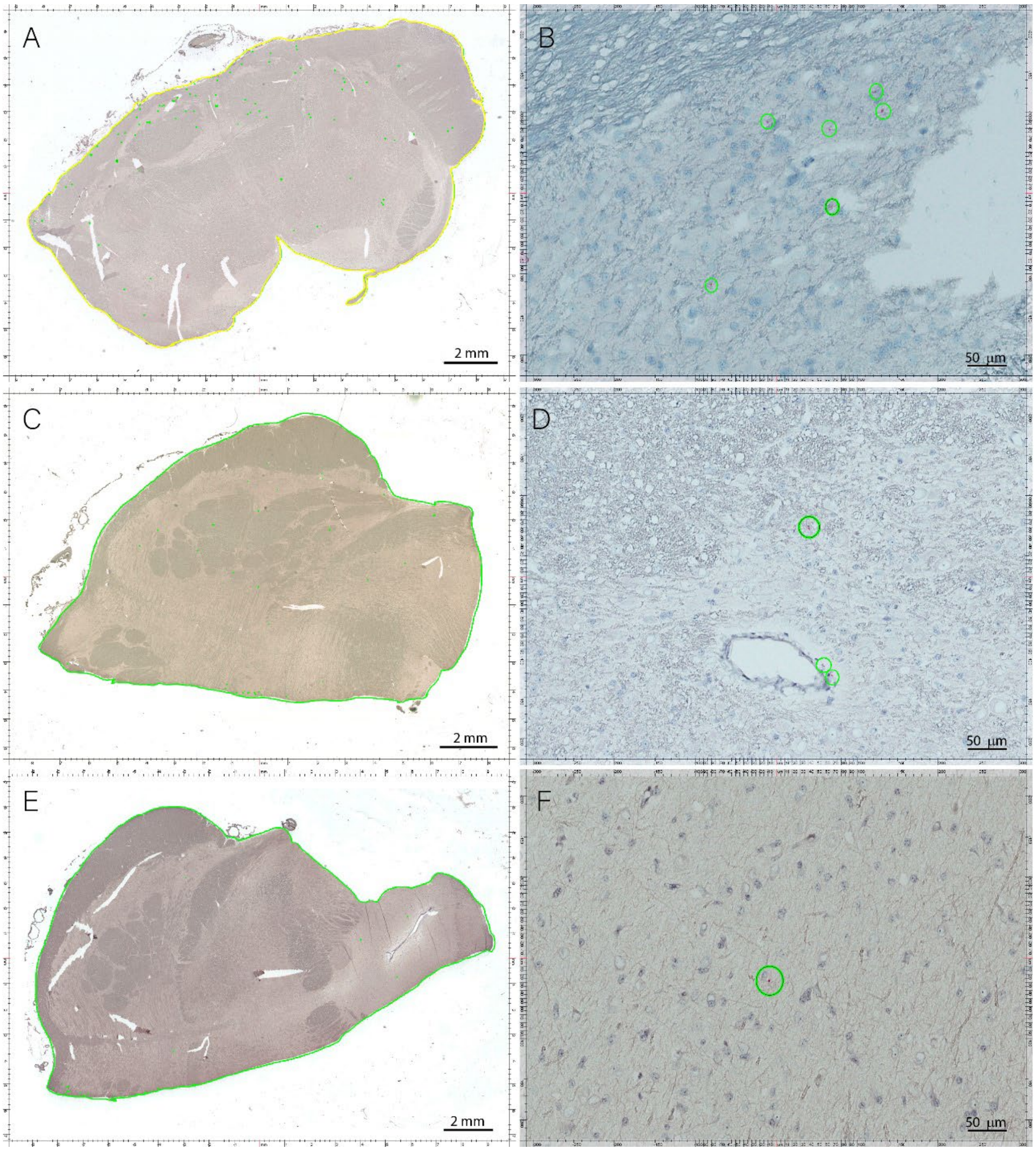

Supplementary Figure 2. SIV RNA detection in the brains of acutely SIVmac251-infected macaques.

Representative midbrain images of SIV RNAscope in situ hybridization from an untreated (A&B; IK30, top), low dose (C&D; 42930, middle), and high dose treated (E&F; 45060, bottom). A,C,E: low magnification; B,D,E: high magnification. SIV RNA+ signals are circled with green circles.

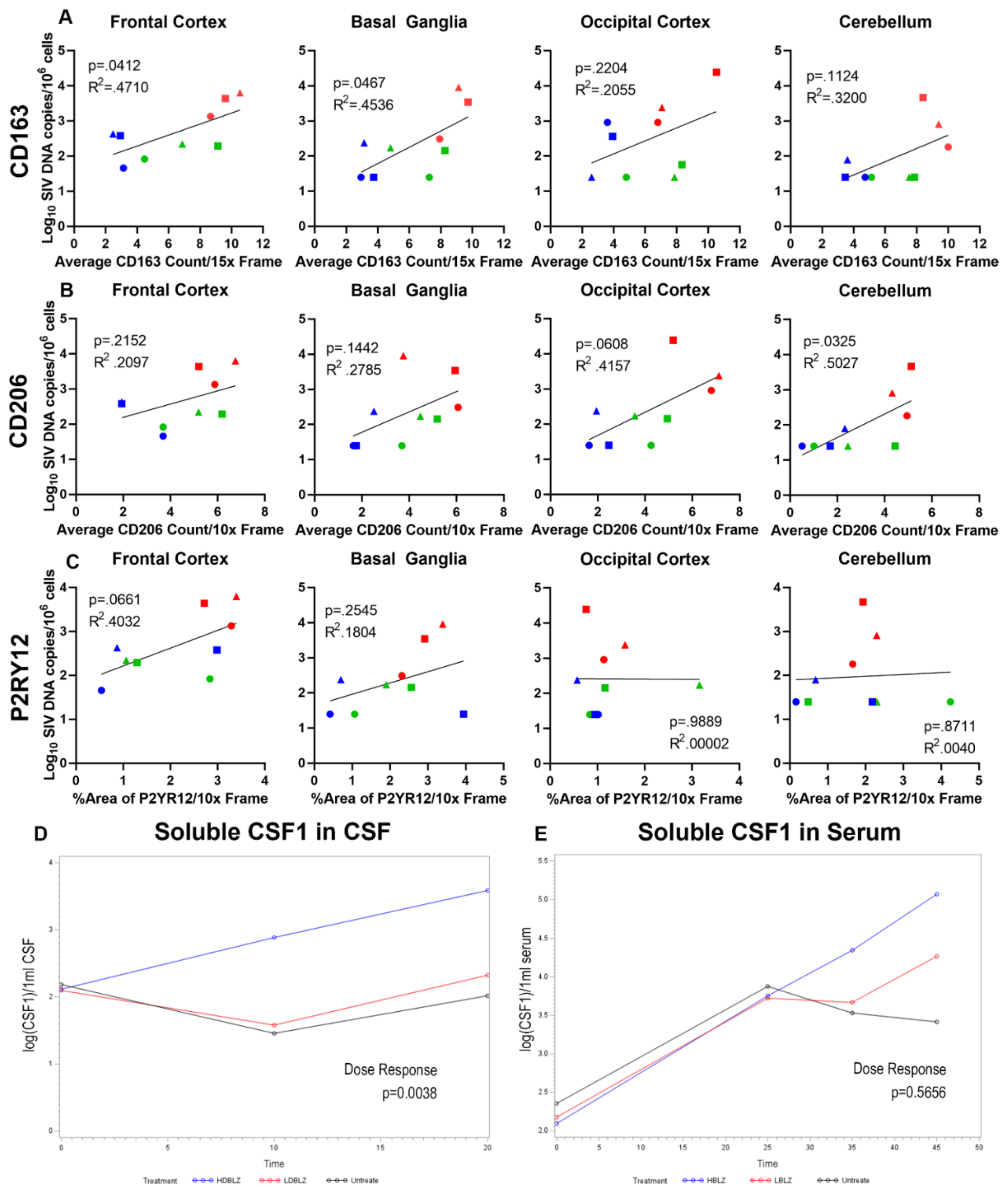

Supplementary Figure 3. Correlations of different macrophages/microglia with levels of SIV DNA in each brain area and circulating CSF1 levels.

Correlations between CD163 (A), CD206 (B), or P2RY12 (C) staining and SIV DNA copies per brain area. Pearson's correlation. The rate of change over time post onset of treatment for soluble CSF1 was measured by Meso Scale Discovery (MSD) in the CSF (D) and serum (E). Dose response curve non-linear regression.

Supplementary Table I Animals used in this study

Graph Symbol	Animal ID	Infection Status	BLZ945 Treatment	Sex	Age
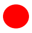	IK30	SIV	Untreated	M	7.08
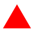	JN80	SIV	Untreated	M	5.45
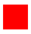	JT99	SIV	Untreated	M	5.27
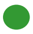	42930	SIV	Low	M	7.87
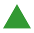	44136	SIV	Low	M	6.04
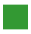	44653	SIV	Low	M	6.86
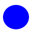	43205	SIV	High	M	8.07
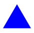	44462	SIV	High	M	6.15
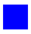	45060	SIV	High	M	5.21
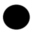	GI84	Uninfected	Untreated	M	4.77
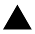	IT02	Uninfected	Untreated	M	4.69
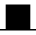	IT24	Uninfected	Untreated	M	5.14

Supplementary Table 2 Antibodies used in this study

Target	Clone	Vendor	Catalogue #	Concentration IHC	Concentration IF
CSF1	2D10	Millipore Sigma	MABF191	0.066	N/A
CSF1R	SP211	Abcam	ab1383316	3.3	N/A
p-CSF1R	49C10	Cell Signaling	3155S	N/A	3.3
CSF1R	B-8	Santa Cruz	sc-46662	N/A	0.2
CD163	EDHu-1	BioRad	MCA1853	0.4	N/A
CD206	5C11	Abnova	H00004360-M02	1.0	N/A
P2RY12	N/A	Millipore Sigma	HPA014518	1.0	N/A
CD68	KP1	Thermo Scientific	MS-397-P	1.0	N/A

Concentration is expressed in μg/mL.

Supplementary Table 3 NanoString differentially regulated genes

High Dose vs Untreated			Low Dose vs Untreated		
**Probe Name**	**Fold Change**	**P value**	**Probe Name**	**Fold Change**	**P value**
CD4	-4.76	0.008545	*JAK3*	-2.5	0.042565
CFB	-3.17	0.036609	IKZF3	-2.34	0.039611
MRC1	-3.04	0.040119	*TGFBI*	-2.27	0.043804
JAK3	-2.66	0.037042	*CDKN1A*	-2.03	0.015682
TGFBI	-2.44	0.032235	MAFK	-1.97	0.046681
PTAFR	-2.44	0.046868	ZBTB16	-1.86	0.031335
ITGB2	-2.28	0.044222	OXER1	-1.86	0.048322
TOLLIP	-2.26	0.043602	*MPPED1*	-1.75	0.033538
CD53	-2.13	0.030642	*MAPK3*	-1.52	0.030222
CSF1R	-1.89	0.010018	B3GAT1	-1.45	0.028037
MPPED1	-1.8	0.037288	*SRC*	-1.25	0.020614
CDKN1A	-1.58	0.037146	*FKBP5*	1.26	0.017803
MAPK3	-1.48	0.039484	*FEZ1*	1.36	0.010506
SRC	-1.28	0.038318	*NFE2L2*	1.44	0.006579
STAT6	1.27	0.030669	*IFIT3*	1.65	0.033314
FEZ1	1.27	0.031124	*IFIT2*	1.68	0.039164
IFNAR1	1.29	0.042178	*PRKCD*	1.93	0.002442
CD83	1.31	0.041617	HLA-DPA1	2.02	0.033107
FKBP5	1.37	0.02369	STAT4	2.43	0.010749
SMPD3	1.39	0.03045	*RPS6KA5*	3.04	0.005284
NFE2L2	1.47	0.004279	IFIT1	3.24	0.019313
ITGB1	1.47	0.010226	BATF	4.97	0.040732
PECAM1	1.47	0.034279	MME	9.69	0.031386
IFIT2	1.58	0.026408			
MX2	1.64	0.045396			
IFIT3	1.68	0.034226			
PRKCD	1.69	0.001525			
OAS2	1.71	0.02428			
DDX58	1.71	0.036356			
CD58	1.72	0.025037			
PSMB8	1.81	0.036571			
IFI44	1.85	0.04201			
CXCL12	1.88	0.037933			
GBP1	2.16	0.043617			
SPP1	2.17	0.042125			
RPS6KA5	3.19	0.000342			

All differentially regulated genes with fold changes greater than +/- 1.25 and P-values < 0.05 for high dose vs untreated (left) and low dose vs untreated (right). Thirteen overlapped genes are presented in italics.

Supplementary Table 4 RNAscope detection of vRNA in 5 brain areas from untreated, low dose, and high dose treated animals.

	**Untreated**	**Low dose**	**High dose**
**Frontal cortex**	0.281 + 0.201	0.050 + 0.034 (*P* = 0.122)	0.045 + 0.012 (*P* = 0.112)
**Temporal cortex**	0.317 + 0.150	0.074 + 0.036 (*P* = 0.053)	0.083 + 0.051 (*P* = 0.063)
**Basal ganglia**	0.277 + 0.094	0.035 + 0.017 (***P* = 0.012**)	0.070 + 0.046 (**P = 0.027**)
**Thalamus**	0.237 + 0.165	0.041 + 0.019 (*P* = 0.110)	0.073 + 0.059 (P = 0.180)
**Midbrain**	0.453 + 0.049	0.159 + 0.079 (***P* = 0.005**)	0.310 + 0.186 (P = 0.267)

The numbers of vRNA per mm2 area was calculated in each brain area using 3 sections per area and averaged. The average + standard deviation (SD) of those average numbers from three animal per group was calculated. *P*-values were determined by Student t-test, unpaired, 2-tailed. For raw data, see Supplementary Table 5 in Excel.
