# Supplementary material for: CSF1R inhibition depletes brain macrophages and reduces brain virus burden in SIV-infected macaques
Source: Brain. 2024 Jul 25;147(9):3059–69. doi: 10.1093/brain/awae153 (PMC11370798; doi:10.1093/brain/awae153)
Supplement: awae153_Supplementary_Data [file awae153_supplementary_data.zip › brain-2023-01175-File008.pdf]

Animal Group	ID	Tissue	vRNA	Area (mm2)	Quant	vRNA	Area (mm2)	Quant	vRNA	Area (mm2)	Quant	AVG
Control	16A512	S2	32	152.52	0.210	33	143.4	0.230	16	156.31	0.102	0.181
		S4A	23	171.25	0.134	77	191.43	0.402	2	182.53	0.011	0.182
		S4B	210	220.12	0.954	41	219.56	0.187	3	215.89	0.014	0.385
		S6B	10	213.19	0.047	20	212.2	0.094	1	208.97	0.005	0.049
		MB1	89	115.2	0.773	38	110.3	0.345	8	104.14	0.077	0.398
	16A465	S2	36	110.67	0.325	7	110.78	0.063	7	117	0.060	0.149
		S4A	203	181.81	1.117	52	197.34	0.264	11	195	0.056	0.479
		S4B	14	144.78	0.097	25	64.2	0.389	28	143	0.196	0.227
		S6B	12	159.56	0.075	106	146.83	0.722	29	227	0.128	0.308
		MB1	117	93.66	1.249	6	93.71	0.064	9	93	0.097	0.470
	16A219	S2	34	94.91	0.358	123	105.89	1.162	3	191.45	0.016	0.512
		S4A	43	189.52	0.227	87	136.39	0.638	–	210.65	0.000	0.288
		S4B	61	173.11	0.352	37	134.87	0.274	5	181.8	0.028	0.218
		S6B	120	172.33	0.696	55	170.46	0.323	8	176.47	0.045	0.355
		MB1	101	122.15	0.827	73	125.84	0.580	8	123.01	0.065	0.491
LD	42930	S2	24	453.63	0.053	46	437.91	0.105	48	436.7	0.110	0.089
		S4A	42	431.71	0.097	94	463.78	0.203	21	448.48	0.047	0.116
		S4B	10	504.99	0.020	22	509.24	0.043	48	518.33	0.093	0.052
		S6B	5	398.74	0.013	2	406.47	0.005	19	397.79	0.048	0.022
		MB1	4	107.36	0.037	37	110.2	0.336	15	109.71	0.137	0.170
	44136	S2	22	448.71	0.049	16	449.17	0.036	1	436.04	0.002	0.029
		S4A	63	487.27	0.129	11	513.85	0.021	3	460.42	0.007	0.052
		S4B	21	528.52	0.040	5	502.47	0.010	1	503.88	0.002	0.017
		S6B	37	387.5	0.095	37	427.25	0.087	–	392.66	0.000	0.061
		MB1	26	139.59	0.186	4	142.01	0.028	1	122.8	0.008	0.074
	44653	S2	17	374.24	0.045	15	384.24	0.039	5	365.09	0.014	0.033
		S4A	30	461.1	0.065	44	463.33	0.095	2	447.63	0.004	0.055
		S4B	23	271.53	0.085	6	275.76	0.022	1	276.13	0.004	0.037
		S6B	28	371.06	0.075	12	363.86	0.033	4	372.36	0.011	0.040
		MB1	94	175.47	0.536	23	176.96	0.130	5	173.86	0.029	0.231
HD	43205	S2	29	318.18	0.091	17	322.35	0.053	5	331.55	0.015	0.053
		S4A	51	439.17	0.116	125	417.61	0.299	–	438.06	0.000	0.138
		S4B	31	498.63	0.062	60	487.59	0.123				0.093
		S6B	14	405.89	0.034	16	409.5	0.039	–	413.37	0.000	0.025
		MB1	227	169.88	1.336	27	164.75	0.164	4	171.5	0.023	0.508
	44462	S2	27	551.83	0.049	23	553.86	0.042	2	531.31	0.004	0.031
		S4A	33	517.09	0.064	29	516.26	0.056	–	490.83	0.000	0.040
		S4B	12	456.03	0.026	11	455.54	0.024	–	478.92	0.000	0.017
		S6B	49	303.63	0.161	73	314.35	0.232	7	307.48	0.023	0.139
		MB1	98	126.26	0.776	9	127.08	0.071	–	116.19	0.000	0.282
	45060	S2	37	560.68	0.066	48	573.35	0.084	–	555.49	0.000	0.050
		S4A	103	539.31	0.191	8	521.67	0.015	1	502.1	0.002	0.069
		S4B	11	446.66	0.025	119	435.29	0.273	2	420.38	0.005	0.101
		S6B	54	364.56	0.148	7	364.65	0.019	–	350.44	0.000	0.056
		MB1	32	125.21	0.256	22	134.54	0.164	–	122.03	0.000	0.140

S2	Control	LD	HD
	0.181	0.089	0.053
	0.149	0.029	0.031
	0.512	0.033	0.050
**AVG**	0.281	0.050	0.045
**SD**	0.201	0.034	0.012
**P**		0.122	0.112

S4A	Control	LD	HD
	0.182	0.116	0.138
	0.479	0.052	0.040
	0.288	0.055	0.069
**AVG**	0.317	0.074	0.083
**SD**	0.150	0.036	0.051
**P**		0.053	0.063

S4B	Control	LD	HD
	0.385	0.052	0.093
	0.227	0.017	0.017
	0.218	0.037	0.101
**AVG**	0.277	0.035	0.070
**SD**	0.094	0.017	0.046
**P**		**0.012**	**0.027**

S6B	Control	LD	HD
	0.049	0.022	0.025
	0.308	0.061	0.139
	0.355	0.040	0.056
**AVG**	0.237	0.041	0.073
**SD**	0.165	0.019	0.059
**P**		0.110	0.180

MB1	Control	LD	HD
	0.398	0.170	0.508
	0.470	0.074	0.282
	0.491	0.231	0.140
**AVG**	0.453	0.159	0.310
**SD**	0.049	0.079	0.186
**P**		**0.005**	0.267
